# Low Electrolytic Conductivity Standards

**DOI:** 10.6028/jres.100.039

**Published:** 1995

**Authors:** Yung Chi Wu, Paula A. Berezansky

**Affiliations:** National Institute of Standards and Technology, Gaithersburg, MD 20899-0001

**Keywords:** benzoic acid, dielectric constant, electrolytic conductivity, low conductivity standard, nonaqueous solvent, Onsager limiting law, potassium chloride

## Abstract

Standards of low electrolytic conductivity were developed to satisfy the demands of the U.S. Navy and American industry for the measurement of high quality water. The criteria for the selection of appropriate solvent and solutes, based on the principles of equivalent conductivity and Onsager’s limiting law, are described. Dilute solutions of potassium chloride and benzoic acid in 30 % *n*-propanol–water have been chosen as standards. The electrolytic conductivity of both sets of these solutions as a function of molality was determined. Solutions of potassium chloride and of benzoic acid are recommended for use as 5 μS/cm, 10 μS/cm, 15 μS/cm, 20 μS/cm, and 25 μS/cm conductivity standards. Solutions prepared from potassium chloride in 30 % *n*-propanol–water have been certified as Standard Reference Materials (SRMs). SRM 3198 and SRM 3199 are certified nominally at 5 μS/cm and 15 μS/cm, respectively, at 25.000 °C.

## 1. Introduction

The monitoring and control of the quality of feed-water and boiler water are necessary for power plants. The generation of steam at high temperature and pressure requires that contaminants be strictly limited to very low levels to prevent corrosion and scaling. Electrolytic conductivity is used as a measure of ionic contaminants in water. The purpose of this investigation is to develop simple, stable reference solutions of low electrolytic conductivity which could serve as standards for laboratory calibration and quality assurance measurements.

Based on the limiting ionic equivalent conductivities of hydrogen ion and hydroxide ion and dissociation constant of water, the theoretical value for the electrolytic conductivity of pure water at 25 °C is 0.055 μS/cm. The absorption of ambient carbon dioxide by the water can cause the electrolytic conductivity to increase by a factor of 10 to 30, depending on the level of carbon dioxide in the atmosphere. In our laboratory, the conductivity of pure water equilibrated with ambient carbon dioxide varies from 0.8 μS/cm to 1.2 μS/cm. Obviously, high quality water will have a very low electrolytic conductivity, that is, in the neighborhood of 1 μS/cm. Moreover, if the water is contaminated with a minute amount of ionic substance, for example, 1 mg/g calcium chloride, the electrolytic conductivity increases to about 3 μS/cm. Hence, low electrolytic conductivity standards in the range of 5 μS/cm to 25 μS/cm are necessary.

## 2. Theory

### 2.1 Electrolytic Conductivity

The electrolytic conductivity^1^ (in S/cm), *κ*, of an electrolyte solution is determined by

$$\kappa =\frac{K_{\text{cell}}}{R},$$ (1)

where *K*_cell_ is the cell constant^2^ (in cm^−1^) of the given cell containing the solution being measured and *R* is the resistance (in Ω) of that solution in that cell.

There is another important quantity for conductivity in the literature, the equivalent conductivity^3^, *Λ* (in S cm^2^/equiv) that is related to *κ* by the following relationship

$$\Lambda =\frac{1000\;{\text{cm}}^{3}}{L}\frac{\kappa }{c},$$ (2)

where *c* is the equivalent concentration^4^ (in equiv/L).

IUPAC recommends the use of equivalent conductivity be discontinued [1]. The use of molar conductivity is preferred. However, the theory behind electrolytic conductivity is based on the equivalent. In order to keep this discussion consistent with theory, we continue to use the terms equivalent and equivalent conductivity. We have shown the equations relating equivalent conductivity and molar conductivity.

The limiting law for *Λ* may be expressed as

$$\Lambda =\Lambda {}^{\circ}-S\sqrt{c},$$ (3)

where *Λ*° is the limiting equivalent conductivity of the electrolyte (*Λ* at infinite dilution) and *S* is the limiting slope of the electrolyte. According to Kohlrausch [2, 3], *Λ*° is the sum of its ionic parts, thus,

$$\Lambda {}^{\circ}=\lambda_{+}^{{}^{\circ}}+\lambda_{-}^{{}^{\circ}},$$ (4)

where *λ*° is the limiting ionic equivalent conductivity of a given ion. Many of the *λ*° in aqueous solution are known and tabulated. For all ions except H^+^ and OH^−^, *λ*° is in the neighborhood of 60 S cm^2^/equiv at 25 °C, so that *Λ*° is typically about 120 S cm^2^/equiv. At low concentration, *Λ*≈*Λ*° [from Eq. (3)], and the combination of Eqs. (2) and (4) yields

$$\kappa =\frac{(\lambda_{+}^{{}^{\circ}}+\lambda_{-}^{{}^{\circ}})c}{\frac{1000\;{\text{cm}}^{3}}{L}}.$$ (5)

If *c* = 10^−4^ equiv/L, then *κ* ≈ 12 μS/cm. However, aqueous solutions tend to absorb carbon dioxide upon exposure to air. Part of the dissolved carbon dioxide (CO_2_) is hydrated and forms carbonic acid (H_2_CO_3_) in water. The carbonic acid then dissociates to hydrogen (H^+^) and bicarbonate (HCO_3_^−^) ions which increase the electrolytic conductivity. The electrolytic conductivity of pure water equilibrated with atmospheric carbon dioxide can range from 0.7 μS/cm to 1.3 μS/cm depending on atmospheric pressure in the particular laboratory, giving a standard uncertainty^5^ ± 0.2 μS/cm. For a 12 μS/cm solution, this amounts to a relative standard uncertainty in the electrolytic conductivity of about ± 2 %. If the electrolytic conductivity requirement is less than 12 μS/cm, the relative standard uncertainty will increase accordingly.

### 2.2 Properties of Nonaqueous and Nonaqueous-Aqueous Mixed Solvents

In order to minimize the relative standard uncertainty of low electrolytic conductivity standards, nonaqueous solvents may be used. Carbon dioxide does not hydrate to form carbonic acid in nonaqueous solvents. In addition, many nonaqueous solvents have lower dielectric constants. Since the dielectric constant is one of the factors which influence ion-pair formation, a decrease in the dielectric constant causes the conductivity to decrease. Table 1 illustrates this point [4].

**Fig. 1:**
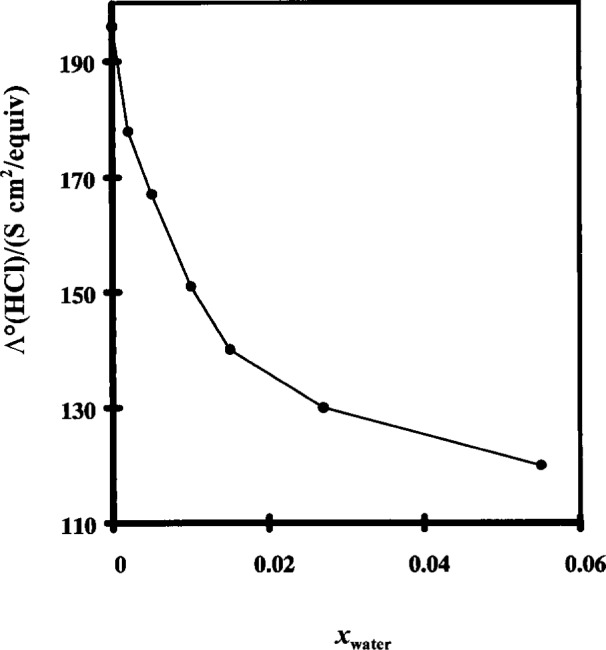
Limiting equialent conductivity of hydrochloric acid in methanol as a function of the mole fraction of water in the solvent.

**Fig. 2:**
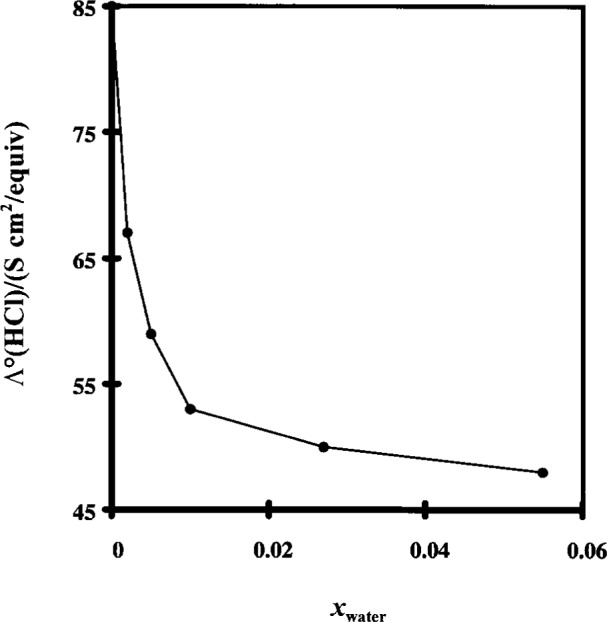
Limiting equivalent conductivity of hydrochloric acid in ethanol as a function of the mole fraction of water in the solvent.

Despite the advantage of inherent low conductivity and low carbon dioxide absorption, the purity of a non-aqueous solvent is difficult to maintain. The amount of moisture in the solvent has a dramatic effect on the corresponding conductivity. This effect is illustrated in Figs. 1 and 2 [4]. It can be seen from these figures that as water is added to methanol or ethanol, the limiting equivalent conductivity of HCl decreases rapidly. The same will be true in *n*-propanol. The limiting equivalent conductivity gradually levels off before increasing again as *Λ*°(HCl) approaches its value in pure water. As little as 0.2 % water in ethanol will decrease *λ* °(HCl) from 84 S cm^2^/equiv to 67 S cm^2^/equiv, a 20 % decrease. Rather than trying to maintain moisture-free alcohol, it is more practical to use a nonaqueous-aqueous mixed solvent to avoid the severity of change in conductivity caused by a small amount of moisture contamination.

The reduction in electrolytic conductivity in a non-aqueous-aqueous mixed solvent is substantial. For example, at 25 °C, *Λ*° (HCl) is 426 S cm^2^/equiv in water and approximately 192 S cm^2^/equiv in a 30 % ethanol-water mixture^6^, a 55% decrease from its value in water [5]! Potassium chloride is a strong electrolyte and should behave similarly to hydrochloric acid.

The dissociation constant of weak electrolytes is also reduced in nonaqueous-aqueous mixed solvents. The dissociation constant of acetic acid, *K*_a_(CH_3_COOH, 25 °C) is 1.75×10^−5^ in water and 1.7×10^−6^ in 20 % EtOH-H_2_O, approximately one-tenth its value in water. Acetic acid and carbonic acid are both weak electrolytes and should behave similarly. If the same reduction is applied to carbonic acid, the *K*_a_(H_2_CO_3_, 25 °C) will decrease from 4.3×10^−7^ in water to 4×10^−8^ in 20 % EtOH-H_2_O. This reduction in *K*_a_(H_2_CO_3_, 25 °C) will reduce the concentration of dissolved hydrogen ion in 20 % EtOH-H_2_O by a factor of three. Combining both the reductions in hydrogen ion conductivity and concentration, a typical fluctuation in the partial pressure of carbon dioxide in the atmosphere will cause the electrolytic conductivity of the nonaqueous-aqueous mixed solvent to fluctuate less than ±0.1 μS/cm, which is negligible.

## 3. Low Conductivity Standards

### 3.1 Criteria for Low Conductivity Standards

A conductivity standard is an electrolyte solution with an accurately known electrolytic conductivity, *κ*. Low conductivity standards are generally used for the calibration of measuring equipment. The cell constant, *K*_cell_, can be determined by using a solution of known *κ* at a measured resistance, *R*, as in Eq. (1). Since any uncertainty in these standard *κ*’s will propagate to *K*_cell_ and thus the *κ* of the measured solution, accuracy is an important requirement. However, the required accuracy depends on the conductivity measuring equipment and the application of these standards. The best relative uncertainty obtainable from the best available conductivity measuring equipment in the U.S. open market today is about ±0.1 %. However, the required uncertainty for water quality control measurements, the recommended application of these standards, is about ±1 % or ±2 %, but may even be a few percent higher. Even if the uncertainty of the measurement is increased to ±5 %, a measured conductivity of 5 μS/cm still has an uncertainty of only ± 0.25 μS/cm. Thus, standards to be used for water quality control do not require high accuracy.

The other requirements for low conductivity standards deal with the materials used to prepare these standards. Materials of sufficient purity must be readily available in order to achieve reproducibility. High purity *n*-propanol (99.9+ %) is available commercially. Deionized water (with no carbon dioxide absorption) with an initial conductivity less than 0.2 μS/cm (resistivity greater than 5 MΩ cm) is readily produced in the laboratory with a commercial deionized water unit. If materials are to be stored, they should be stable. Following normal laboratory procedures and precautions, the materials should not be hazardous to health or to the environment so that they can be easily prepared without special training.

### 3.2 Selection of Materials

Based on the principles discussed above, 30 % *n*-PrOH−H_2_O is employed as a solvent. A significant decrease in electrolytic conductivity is expected in this mixed solvent. Similarly, it is likely that the dissociation constant for weak electrolytes in this solvent would also be significantly reduced from its value in water. Because of its lower volatility, *n*-propanol is chosen over methanol and ethanol so that this solvent mixture will provide better stability. Distilled water and high purity (99.9 %) *n*-propanol are easily obtainable. Variation in the amount of carbon dioxide absorbed in the 30 % *n*-PrOH−H_2_O should be low as discussed in Sec. 2.2.

The electrolytes selected were potassium chloride (KCl) and benzoic acid (C_6_H_5_COOH). Both of these materials are solid and are soluble in 30 % *n*-PrOH−H_2_O. The standard solutions can be easily prepared gravimetrically. Both solutes are readily available in high purity and are issued as Standards Reference Materials (SRMs).

The molality of these two electrolyte solutions can be estimated and prepared to blanket the range of electrolytic conductivities of interest. The molalities required for electrolytic conductivities less than 30 μS/cm are less than 0.0005 mol/kg for potassium chloride and less than 0.005 mol/kg for benzoic acid.

## 4. Experimental

### 4.1 Apparatus

The apparatus used for these experiments consists of a constant-temperature oil bath, an ac bridge with a frequency generator and a detector, and Daggett-type cells with cell constants of 0.13965 cm^−1^ and 0.05702 cm^−1^. All have been described in other publications [5, 6].

### 4.2 Materials

SRM 999 Potassium Chloride and SRM 350a Benzoic Acid were used as solutes to prepare the solutions. Fisher^7^ reagent grade *n*-propanol (assay 99.9 %) was mixed with deionized filtered (0.22 μm) water (initial electrolytic conductivity less than 0.2 μS/cm) to prepare 30 % *n*-PrOH−H_2_O to be used as the solvent. The materials were used as received without further purification. All the solutions were prepared gravimetrically at the molality (SI unit mol/kg) specified. Buoyancy corrections were not applied when preparing these solutions because they are insignificant at the uncertainty required in this application.

### 4.3 Procedure

The electrolytic conductivity measurements of potassium chloride and benzoic acid solutions were performed at 25.000 °C ± 0.002 °C. The resistance of each solution in a particular cell was measured and used to calculate the conductivity from Eq. (1). A more detailed explanation of these measurements may be found in previous publications [7, 8]. Each solution was measured at the time of preparation. Some solutions were measured a second time after allowing the solution to sit for approximately two months to determine the stability of the solutions. The temperature coefficients of the electrolytic conductivity at 25 °C for solutions of potassium chloride and benzoic acid were evaluated by the measurement of a potassium chloride solution with a molality of 0.000400 mol/kg and a benzoic acid solution with a molality of 0.00500 mol/kg at 20 °C, 25 °C, and 30 °C.

### 4.4 Results

The electrolytic conductivity of potassium chloride and benzoic acid in 30 % *n*-PrOH−H_2_O was calculated from the measured resistance of each solution and is listed in Tables 2 and 3. The conductivity at various temperatures of one solution of each solute is listed in Table 4. The values from Tables 2 and 3 at the time of preparation were smoothed by regression analysis to give the following relationships at 25 °C

$$\kappa (\text{KCl})=0.35+6.38\times 10^{4}m$$ (6)

$$\kappa (C_{6}H_{5}\text{COOH})=0.04+376\;m^{1/2}$$ (7)

where κ is given in μS/cm and *m* is given in mol/kg. The uncertainty of *κ*(KCl) is ± 0.1 μS/cm and the uncertainty of *κ*(C_6_H_5_COOH) is ± 0.2 μS/cm. The molalities for given values of electrolytic conductivity are computed and listed in Table 5.

### 4.5 Uncertainty

There are five main sources of uncertainty contributing to the expanded uncertainty^8^ of the conductivity measurements listed in Tables 2, 3, and 4. The uncertainty from random effects is found from the standard deviation of the mean of several measurements of the same solution at the same time (Type A evaluation). This standard uncertainty is less than 60.01 μS/cm. There are four main sources of uncertainty from systematic effects and are obtained from Type B evaluations. The standard uncertainty of the cell constant is ± 0.0002*K*_cell_/3^1/2^, and translates to a standard uncertainty in the conductivity of ± 0.00012*κ*. The standard uncertainty of the resistance measurements is ±0.00005*R*/3^1/2^, giving a standard uncertainty of ±0.00006*κ*. The standard uncertainty of the temperature of the oil bath is ±0.002 °C/3^1/2^. Assuming a temperature coefficient for the solution of 3 %/°C, the standard uncertainty in the electrolytic conductivity is ±0.000035*κ*. The standard uncertainty due to carbon dioxide absorption is ±0.06 μS/cm as given in Sec. 2.2. The expanded uncertainty of our electrolytic conductivity measurements listed in Tables 2, 3, and 4 is estimated to be ±0.12 μS/cm calculated according to the CIPM approach [8].

There is an additional component of uncertainty for the electrolytic conductivity values listed in Table 5. The standard uncertainty contributed from the regression is no greater than ± 0.2 μS/cm. This value should be combined quadratically with the uncertainty of the measurement to arrive at the expanded uncertainty for the standards listed in Table 5. These values of electrolytic conductivity and uncertainty are listed in Table 6 and are only valid for solutions prepared from the materials specified in Sec. 4.2.

The expanded uncertainty of most field measurements of conductivity is generally much greater than the expanded uncertainty discussed above concerning measurements performed in our laboratory. The major component of uncertainty in field measurements is from the standard uncertainty of the temperature of the measurement. The standard uncertainty in the conductivity is equal to approximately (± 3 %/°C)*κu*_t_ where *u*_t_ is the standard uncertainty of the temperature. The estimated uncertainties of the standards listed in Table 5, prepared from the materials specified in Sec. 4.2, with a temperature stability of ± 1 °C are listed in Table 6.

Because the uncertainties associated with stored solutions (including those that depend on the type of bottle used, the tightness of the cap, the headspace volume in the bottle, as well as the environment in which the bottle is stored) in laboratories other than our own cannot be estimated, the above total uncertainty is valid only for a solution at the time of preparation.

## 5. Conclusion

In view of the results shown above, these two sets of solutions have met the criteria for electrolytic conductivity standards described in Sec. 3.1. Therefore, they may be used as low electrolytic conductivity standards.

Highest accuracy can be achieved by performing electrolytic conductivity measurements in a constant temperature bath. Because the temperature coefficient is only 2 %/°C to 3 %/°C, the electrolytic conductivity of these standards in an air-conditioned laboratory at typical room temperature (held stable to ± 1 °C) will still have a relative expanded uncertainty of approximately ± 4 %.

The recommended standards are easy to prepare. They are fairly stable, as seen in Tables 2 and 3, and can be used for at least a month after preparation provided the containers are kept tightly capped. The molality and mass fraction for the nominal electrolytic conductivity values of the standard are given in Table 5. The estimated uncertainties of these standards are listed in Table 6.

## Figures and Tables

**Table 1:** Limiting equivalent conductivity of selected electrolytes in various solvents at 25 °C

Solvent	Dielectric constant	*Λ*°/(S cm^2^/equiv)		
		HCl	NaCl	KCl
Water	78.3	426	126.5	150
Methanol	32.6		98	105
Ethanol	24.3	84	42.5	45.4
*n*-Propanol	20.4	30		

**Table 2:** Electrolytic conductivity of KCl in 30 % *n*-PrOH−H_2_O at 25 °C

*m*_KCl_/(10^−3^ mol/kg)	*κ* /(μS/cm)		Change
	1st measurement	2nd measurement	
0.100	6.79	6.86	+ 1 %
0.300	19.60		
0.400	25.76	25.89	+ 0.5%
0.500	32.18	32.32	+ 0.5%

**Table 3:** Elecrolytic conductivity of benzoic acid in 30 % *n*−PrOH−H_2_O at 25 °C

$m_{C_{6}H_{5}\mathrm{COOH}}/(10^{-3}\text{mol}/\text{kg})$	*κ*/(μS/cm)		Change
	1st measurement	2nd measurement	
0.100	3.46		
0.300	6.30		
0.600	9.05		
1.00	11.76		
4.368	24.95	25.11	+ 0.6 %
5.00	26.83	27.00	+ 0.6 %

**Table 4:** Temperature coefficients of electrolytic conductivity for selected dilute KCl and C_6_H_5_COOH solutions in 30 % *n*-PrOH−H_2_O

Electrolyte	*m*/(mol/kg)	*κ*/(μS/cm)			(Δ*κ*/*κ*)/Δ*t* at 25 °C
		20 °C	25 °C	30 °C	
KCl	0.000400	22.11	25.76	29.57	2.9%/°C
C_6_H_5_COOH	0.00500	23.52	27.00	30.59	2.6 %/°C

**Table 5:** Molality and mass fraction (given in grams solute per kilogram solution) of low conductivity standards in 30 % *n*−PrOH−H_2_O

*κ* /(μS/cm)	KCl		C_6_H_5_COOH	
	*m* /(10^−3^ mol/kg)	*w* /(g/kg)	*m* /((10^−3^ mol/kg)	*w* /(g/kg)
5.00	0.0729	0.00543	0.174	0.0213
10.0	0.151	0.0113	0.702	0.0857
15.0	0.230	0.0171	1.58	0.193
20.0	0.308	0.0230	2.82	0.344
25.0	0.386	0.0288	4.41	0.538

**Table 6:** Estimated expanded uncertainty and relative uncertainty of conductivity standards at various temperature stabilities

*κ* /(μS/cm)	Stable to ±0.002 °C		Stable to ±1 °C	
	Total uncertainty/(μS/cm)	Relative uncertainty	Total uncertainty/(μS/cm)	Relative uncertainty
5.00	0.23	4.7 %	0.29	5.8 %
10.0	0.23	2.3 %	0.42	4.2 %
15.0	0.23	1.6 %	0.57	3.8 %
20.0	0.23	1.2 %	0.73	3.6 %
25.0	0.23	0.94 %	0.90	3.6 %

## Footnotes

1 Electrolytic conductivity (formerly called specific conductance) is commonly expressed in μS/cm. The SI unit of conductance is the siemens, S, and is equivalent to an inverse ohm, Ω^−1^.

2 The symbol *G* is often used for the cell constant. IUPAC recommends the use of the symbol *G* for conductance and *K*_cell_ for cell constant.

3 Equivalent conductivity is equal to *Λ*_m_/*ν*_+_*z*_+_ where *ν*_+_ is the number of cations of charge number *z*_+_ produced in the dissociation of a salt “molecule” of a given type, of which *ν*_+_*z*_+_ = *ν*_−_*z*_−_ and *Λ*_m_ is the molar conductivity [1].

4 Equivalent concentration is equal to *ν*_+_*z*_+_*c*_m_ where *c*_m_ is the concentration.

5 The standard uncertainty is calculated from the lower limit, *a*_−_, and upper limit, *a*_+_, of a value by (*a*_+_+*a*_−_) /((2)(3^1/2^)) where 3^1/2^ is used to model the value to a rectangular probability distribution.

6 This mixed solvent is referred to being a “30 % ethanol-water mixture” or “30 % EtOH-H_2_O.” The 30 % is given in “weight percent” so that in every 100 g of solvent, there are 30 g ethanol and 70 g water.

7 Certain commercial equipment, instruments, or materials are identified in this paper to foster understanding. Such identification does not imply recommendation or endorsement by the National Institute of Standards and Technology, nor does it imply that the materials or equipment identified are necessarily the best available for the purpose.

8 The expanded uncertainty is intended to give a 2 standard deviation estimate where the coverage factor, *k* = 2 [9].
